# A segmentation-informed deep learning framework to register dynamic two-dimensional magnetic resonance images of the vocal tract during speech

**DOI:** 10.1016/j.bspc.2022.104290

**Published:** 2023-02

**Authors:** Matthieu Ruthven, Marc E. Miquel, Andrew P. King

**Affiliations:** a Clinical Physics, Barts Health NHS Trust, West Smithfield, London EC1A 7BE, United Kingdom; b School of Biomedical Engineering & Imaging Sciences, King’s College London, King’s Health Partners, St Thomas’ Hospital, London SE1 7EH, United Kingdom; c Digital Environment Research Institute (DERI), Empire House, 67-75 New Road, Queen Mary University of London, London E1 1HH, United Kingdom; d Advanced Cardiovascular Imaging, Barts NIHR BRC, Queen Mary University of London, London EC1M 6BQ, United Kingdom

**Keywords:** Convolutional neural networks, Registration, Segmentation, Dynamic magnetic resonance imaging, Speech, Articulators

## Abstract

**Objective:**

Dynamic magnetic resonance (MR) imaging enables visualisation of articulators during speech. There is growing interest in quantifying articulator motion in two-dimensional MR images of the vocal tract, to better understand speech production and potentially inform patient management decisions. Image registration is an established way to achieve this quantification. Recently, segmentation-informed deformable registration frameworks have been developed and have achieved state-of-the-art accuracy. This work aims to adapt such a framework and optimise it for estimating displacement fields between dynamic two-dimensional MR images of the vocal tract during speech.

**Methods:**

A deep-learning-based registration framework was developed and compared with current state-of-the-art registration methods and frameworks (two traditional methods and three deep-learning-based frameworks, two of which are segmentation informed). The accuracy of the methods and frameworks was evaluated using the Dice coefficient (DSC), average surface distance (ASD) and a metric based on velopharyngeal closure. The metric evaluated if the fields captured a clinically relevant and quantifiable aspect of articulator motion.

**Results:**

The segmentation-informed frameworks achieved higher DSCs and lower ASDs and captured more velopharyngeal closures than the traditional methods and the framework that was not segmentation informed. All segmentation-informed frameworks achieved similar DSCs and ASDs. However, the proposed framework captured the most velopharyngeal closures.

**Conclusions:**

A framework was successfully developed and found to more accurately estimate articulator motion than five current state-of-the-art methods and frameworks.

**Significance:**

The first deep-learning-based framework specifically for registering dynamic two-dimensional MR images of the vocal tract during speech has been developed and evaluated.

## Highlights

• A segmentation-informed deformable registration framework was developed.
• It estimates displacement fields between dynamic 2D MR images of the vocal tract.
• Developed to facilitate quantitative analysis of articulator motion during speech.
• Intended for use in clinical and non-clinical studies of speech.
• The framework was evaluated using a clinically relevant metric.

## 1 Introduction

### 1.1 Dynamic imaging of speech

Human speech production is a complex process involving the coordinated motion of speech organs, or articulators, including the tongue and soft palate.

Dynamic imaging of the vocal tract enables visualisation of articulators during speech, thus providing information about their position, size, shape and motion. In a research context, this information has helped to increase our understanding of speech production [1], [2], [3], [4], [5], [6], while in a clinical context, this information aids the management of patients with speech problems by informing treatment decisions [7], [8], [9].

Dynamic magnetic resonance imaging (MRI) has several advantages over other dynamic imaging techniques: it is non-invasive, can image planes of any orientation and does not use ionising radiation. These advantages have resulted in its increasing use to visualise the articulators during speech [1], [2], [3], [4], [5], [6], [10], [11], [12], [13]. Dynamic MRI is also beginning to be used in the clinical assessment of speech, for example the speech of patients with velopharyngeal insufficiency [14], [15], [16], [17], [18], [19], patients following glossectomy [20], [21] and people who stutter [22].

### 1.2 Articulator motion and its quantification

During speech, the articulators move in a complex manner. As well as changing shape and position, they come into contact and separate from each other and anatomical structures such as the pharyngeal wall. In normal speech the soft palate often comes into contact with the pharyngeal wall, a phenomenon known as velopharyngeal closure. In patients with certain types of speech problems, information about the motion of the soft palate, including whether or not velopharyngeal closure occurs, helps clinicians to diagnose the causes of speech problems and therefore informs patient management decisions [7], [8], [9], [15], [16], [23].

Dynamic MRI of speech involves acquiring series of usually two-dimensional (2D) images of the vocal tract. There is increasing interest in automatic quantification of articulator motion in these series, for example to facilitate analysis of articulator motion before and after treatment in patients with speech problems. An established way to automatically quantify complex motion in an image series is by using a deformable image registration method to estimate displacement fields between the images. However, additional information is required to identify which regions of the displacement fields correspond to different articulators. This information can automatically be provided using segmentation methods.

### 1.3 Related work

Traditional deformable registration methods establish nonlinear spatial correspondences (usually displacement vector fields) between two images by iteratively optimising a cost function [24]. Many different types of methods have been developed and used to register a wide variety of medical images [24]. Well-established methods include free-form deformations (FFD) [25], demons [26], discrete methods [27] and their extensions such as [28], [29]. Most traditional deformable registration methods are designed to estimate smooth and continuous displacement fields. However, such fields cannot accurately capture certain types of motion such as organs sliding past each other or organs coming into contact and then separating from each other. Instead, displacement fields with discontinuities are required to capture these types of motion. While several methods [30], [31], [32], [33], [34] have been developed to capture the former type of motion, only one of these [33] can capture the latter type. This method would be particularly suitable for capturing the motion of the articulators during speech, however, unfortunately there is no publicly available implementation of it.

Recently, inspired by the successes of deep-learning-based methods in other medical image analysis tasks, researchers have developed deep-learning-based deformable registration methods [35], [36], [37], [38], [39], [40]. The latest methods [35], [36], [37], [38] are unsupervised or weakly-supervised and consist of convolutional neural networks (CNNs) for estimating displacement fields between images and spatial transformers [41] for transforming images and/or segmentations according to the estimated displacement fields. These methods have achieved state-of-the-art accuracy in the registration of magnetic resonance (MR) images of organs including the heart [35], [36] and brain [37], [38].

Registration and segmentation can be related tasks, and there is increasing evidence that including segmentation information during the training of a registration CNN results in more accurate motion estimates [37], [42], [43], [44], [45], [46], [47], [48], [49], [50], [51]. Inclusion of such information is typically achieved by including region-overlap-based terms such as the Dice coefficient (DSC) in the CNN loss function. Joint registration and segmentation frameworks [42], [44], [45], [46], [47], [48] have been developed as well as “segmentation-informed” registration frameworks such as VoxelMorph [37]. In fact, VoxelMorph can be trained in two ways: (i) using only the estimated displacement fields and the fixed and transformed moving images in the loss function, and (ii) in a segmentation-informed manner, where fixed and transformed moving segmentations are also used.

Segmentation information has also been included in the registration process in two other ways. The first approach is to use segmentations to modify the appearance of the images, in order to optimise the images for the registration task [49], [50], [51]. In this approach, the images are modified before being used as inputs to the registration CNNs either by multiplying them by binary segmentations [49], [50] or by using a fully convolutional image transformer network whose loss function includes a region-overlap-based term [51]. The second approach is to use segmentations as well as images as inputs to the registration CNN [43]. The rationale for inputting segmentations, even if these are estimates rather than ground-truths, is that they provide information about the positions of anatomical features in the images and would therefore help the CNN to estimate more accurate displacement fields.

Similarly to traditional deformable registration methods, currently the majority of deep-learning-based methods are designed to estimate smooth and continuous displacement fields. Two methods have been developed to estimate displacement fields with discontinuities [50], [52]. [52] is designed to capture sliding motion only, while [50] is designed to capture cardiac motion and its suitability for capturing motion where organs come into contact and then separate from each other has not yet been investigated.

Several studies have used traditional deformable registration methods to estimate displacement fields between images in series of dynamic 2D MR images of the vocal tract during speech [11], [12], [13]. In [11], [12] the diffeomorphic demons method [28] was used to estimate displacement fields, while in [13] a registration method based on optical flow [53] was used. In [12], [13], images showing the tongue and soft palate in contact were registered to images showing the tongue and soft palate not in contact. However, neither study evaluated nor discussed if the registration methods captured this change in contact. No prior work has investigated segmentation-informed registration of MR images of the vocal tract.

Several methods to segment articulators in dynamic 2D MR images of the vocal tract during speech have been developed [54], [55], [56], [57], [58], [59], [60], [61], [62], [63], [64]. However, only one of these fully segments several groups of articulators in the images [56].

### 1.4 Displacement field accuracy evaluation

To accurately represent soft palate motion, displacement fields estimated by deformable registration methods must capture any velopharyngeal closures that occur. However, standard metrics such as region-overlap-based terms do not evaluate this.

A metric based on velopharyngeal closure has been proposed and used to evaluate the accuracy of a method to segment dynamic 2D MR images of the vocal tract during speech [56]. This metric quantifies how many of the velopharyngeal closures in the ground-truth (GT) segmentations occur in the estimated segmentations, and is calculated by comparing corresponding consecutive segmentations in the two series. It could also be used to evaluate the accuracy of a registration method. In this case, the metric would be calculated by comparing the GT segmentations of the fixed images with the transformed GT segmentations of the moving images.

### 1.5 Contributions

This work includes two contributions. First, it adapts a current state-of-the-art segmentation-informed deep-learning-based deformable registration framework to optimise it for estimating displacement fields between dynamic 2D MR images of the vocal tract during speech. This is the first time that segmentation-informed registration has been used for this application. Second, this work uses for the first time a metric based on a quantifiable and clinically relevant aspect of articulator motion (velopharyngeal closure) to evaluate the accuracy of these displacement fields.

## 2 Methods

### 2.1 Proposed deformable registration framework

**Fig. 1:**
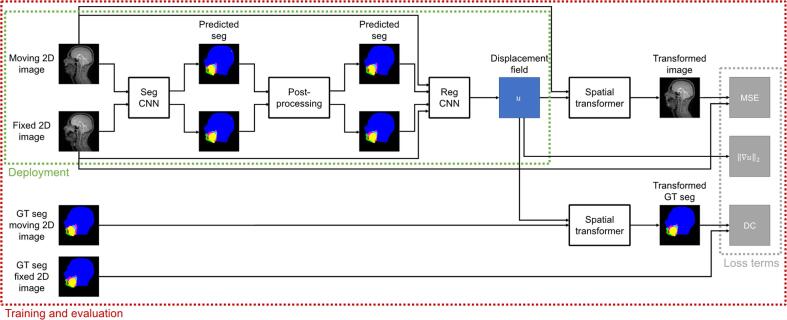
An overview of the proposed framework. A pair of dynamic two-dimensional (2D) magnetic resonance images pass through the framework as follows. First, the image pair are used as inputs to a convolutional neural network (CNN) which estimates segmentations of six different anatomical features in the images. Second, the segmentations are post-processed to remove anatomically impossible regions. Third, the image pair and post-processed segmentations are used as inputs to a registration CNN which estimates a displacement field to align the moving image to the fixed image. Fourth, the moving image and displacement field are used as inputs to a spatial transformer to transform the moving image. During training and evaluation, the spatial transformer is also used to transform the ground-truth (GT) segmentations of the moving image. The red boundary contains the parts of the framework used during training and evaluation, while the green boundary contains the parts used during deployment. The grey boundary contains the terms in the loss function used to train the framework. (For interpretation of the references to colour in this figure legend, the reader is referred to the web version of this article.)

**Fig. 2:**
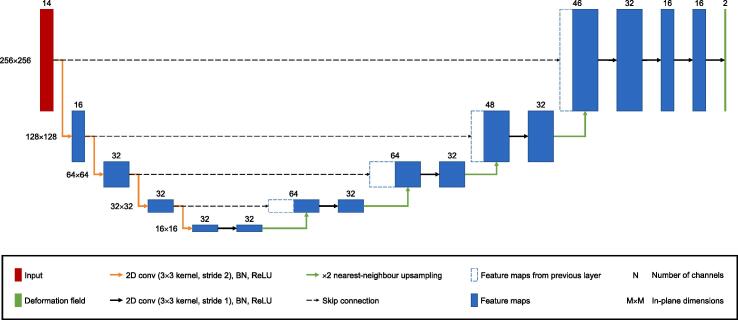
The architecture of the registration convolutional neural network in the proposed framework (i.e. the Reg CNN box in Fig. 1). When input with a pair of dynamic two-dimensional (2D) magnetic resonance images of the vocal tract and segmentations of six different anatomical features in the pair, the network estimates a displacement field to align one of the images to the other. The network has 14 input channels: two for the image pair, six for the segmentations of the fixed image and six more for the segmentations of the moving image. The network output has 2 channels: one for displacements in the x-direction and another for displacements in the y-direction. The outputs of each 2D convolution (conv) are batch normalised. Following batch normalisation (BN), the outputs are passed through a rectified linear unit (ReLU).

Fig. 1 shows an overview of the proposed framework. Given a pair of images from a series of dynamic 2D MR images of the vocal tract, the framework will estimate a displacement field to align the moving image to the fixed image. The framework is based upon the segmentation-informed VoxelMorph framework [37] but features two adaptations. First, it includes a method to segment the images. Second, segmentations as well as images are used as inputs to the registration CNN, in the same manner as the framework of Chen et al. [43]. Fig. 2 shows the architecture of the registration CNN. Since six anatomical features are segmented in the images, the registration CNN has 14 input channels (two for the 2D fixed and moving images, 12 for the 2D fixed and moving segmentations), while the registration CNN of VoxelMorph only has two (for the fixed and moving images). The publicly available implementation of VoxelMorph is designed to allow either 2D or 3D images to be used as inputs to the registration CNN.

The proposed framework includes a deep-learning-based method to estimate segmentations of the following six anatomical features in the image pair: the head, soft palate, jaw, tongue, vocal tract and tooth space. This method is described in [56] and consists of two steps. First, segmentations of the six anatomical features in the image pair are estimated using a pre-trained CNN. Second, a connected-component-based post-processing step is performed to remove anatomically impossible regions from the segmentations. For full information about the segmentation method, the reader is referred to [56].

Like the VoxelMorph frameworks, the proposed framework includes a spatial transformer to transform an image or segmentation according to an estimated displacement field. The spatial transformer is required for framework training and evaluation, but not for framework deployment.

### 2.2 Framework implementation, training and evaluation

The segmentation method used in the framework had been trained separately in the way described in [56]. The framework was trained using the same train/validation/test dataset split as the segmentation method. The framework was implemented in PyTorch 1.7.1 [65] and trained for 200 epochs. In each epoch, every image in the training dataset was used once as the fixed image. Each fixed image was randomly paired with another image of the same subject. Each mini-batch consisted of four image pairs. Segmentations of these images were estimated using the segmentation method. The images and estimated segmentations were then used as inputs to the registration CNN. During training and evaluation, GT segmentations of the images were transformed according to the displacement fields estimated by the registration CNN. The Adam optimiser [66] with$\beta_{1}$ = 0.9, $\beta_{2}$=0.999 and ε = 1e-8 was used during training. Data augmentation consisting of random translations, rotations, cropping and rescaling was performed to increase the size of the training dataset by a factor of four. More information about the augmentations is provided in Section 2.3 of [56]. During framework evaluation, every image in the testing dataset was used as the fixed image. Each image was paired with the reference image of the dataset.

### 2.3 Loss function

The proposed framework was trained using the same loss function as the segmentation-informed VoxelMorph framework. This loss function consisted of three terms: a mean squared error (MSE) term; an L_2_ regularisation of the spatial gradients of the displacement field (u) term and a DSC term. The full loss function was:

(1) $$L=MSE+\lambda {\left\|\nabla u\right\|}_{2}-\gamma DSC$$

where λ and γ are loss weighting terms.

## 3 Experiments

### 3.1 Data

#### 3.1.1 Magnetic resonance images

**Fig. 3:**
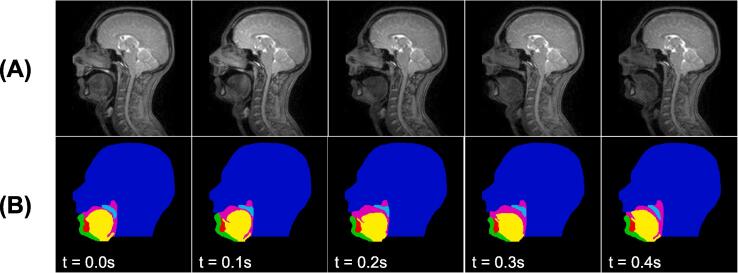
Five consecutive images from one of the series of dynamic two-dimensional magnetic resonance images (A) and ground-truth segmentations of the images (B). The ground-truth segmentations are of the head (dark blue), soft palate (light blue), jaw (green), tongue (yellow), vocal tract (pink) and tooth space (red). t indicates time in seconds. (For interpretation of the references to colour in this figure legend, the reader is referred to the web version of this article.)

Five MR image series of different healthy subjects (two females, three males; age range 24–28 years) were used in this work. All subjects spoke English fluently, had no recent history of speech or language disorders and were imaged in a supine position while counting from one to ten. Imaging was performed using a 3.0 T TX Achieva MRI scanner and a 16-channel neurovascular coil (both Philips Healthcare, Best, the Netherlands) and a fast low-angle shot pulse sequence. Series of 2D images of a 300 × 230 × 10 mm^3^ (256 × 256 matrix) midsagittal slice of the head were acquired at 10 frames per second. The series consisted of 105, 71, 71, 78 and 67 images respectively. Each series was normalised with respect to its maximum and minimum pixel intensities so that the intensities were between 0 and 1. Fig. 3A shows example images. In each series, the first image that met the following criteria was manually chosen to be the reference image:

1. Upper and lower lips not in contact.
2. Tongue not in contact with roof of mouth or soft palate.
3. Soft palate not in contact with pharyngeal wall.

**Fig. 4:**
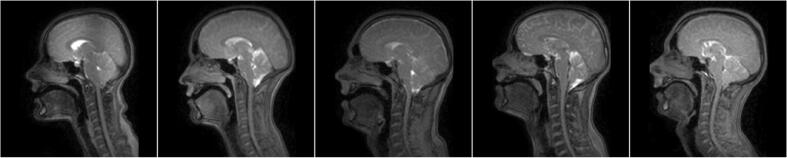
The reference image in each of the five series of dynamic two-dimensional magnetic resonance images. During framework evaluation, these images were used as the moving image for registration purposes.

Fig. 4 shows the reference images. During framework evaluation, these images were used as the moving image for registration purposes.

#### 3.1.2 Ground-truth labels and segmentations

Each image has a label indicating if it shows velopharyngeal closure and also GT segmentations of the following six anatomical features: the head (including the upper lip and hard palate), soft palate, jaw (including the lower lip), tongue (including the epiglottis), vocal tract and tooth space (lower incisor only). More information about the labelling and GT segmentation creation processes is provided in Section 2.2 of [56]. Fig. 3B shows example GT segmentations.

### 3.2 Displacement field accuracy evaluation

Estimated displacement field accuracy was evaluated by transforming moving GT segmentations using the displacement fields and then comparing these with fixed GT segmentations using three metrics, as described below.

#### 3.2.1 Dice coefficient and average surface distance

The DSC was used to quantify the overlap of corresponding features in the fixed and transformed moving GT segmentations, while the average surface distance (ASD) was used to quantify the average discrepancy between pixels at the surfaces of corresponding features. Six values of each metric were calculated per moving segmentation: one value per class.

#### 3.2.2 True velopharyngeal closures

The third metric evaluates if velopharyngeal closures are captured by the displacement fields. The number of true velopharyngeal closures captured by the displacement fields was calculated in the following way. First, transformed moving GT segmentations were automatically labelled as showing velopharyngeal closure or not. This enabled the velopharyngeal closures in a series of segmentations to be represented as a series of binary values (one for each frame) with zero indicating no velopharyngeal closure and one indicating velopharyngeal closure. Second, the binary series of the fixed and transformed moving GT segmentations was automatically compared. A velopharyngeal closure was considered to be captured correctly if a series of ones in both binary series overlapped. The software to label segmentations and create and compare binary series was developed in-house and implemented using MATLAB 2019b (MathWorks, Natick, MA). The software determined if a segmentation frame showed velopharyngeal closure by identifying if three or more posterior “soft palate” pixels in the frame were in contact with “head” pixels.

### 3.3 Five-fold cross-validation

A five-fold cross-validation was carried out to evaluate the generalisability of the framework. A different image series was left out in each fold. Hyperparameter optimisation was performed as part of the cross-validation, by carrying out a nested cross-validation for each main cross-validation fold. The nested cross-validations were four-fold cross-validations where each of the remaining four image series were left out once. In each nested cross-validation fold, eight combinations of learning rates and loss term weightings (given in Supplementary Materials Table 1) were evaluated. The optimal hyperparameter combination was identified by comparing the number of true velopharyngeal closures captured by the displacement fields estimated for the left-out image series of the nested cross-validation. The combination that resulted in the capture of the largest number of true velopharyngeal closures was chosen as the optimal hyperparameter combination. Once the optimal combination had been identified for a main cross-validation fold, these hyperparameters were used to train the framework. In each main cross-validation fold, the framework was trained using all the image series except the left-out image series for that fold, and then evaluated using the left-out image series.

### 3.4 Comparison with state-of-the-art methods and frameworks

The proposed framework was benchmarked against five current state-of-the-art deformable registration methods and frameworks: two traditional methods and three frameworks. The traditional methods were FFD [25] and a segmentation-informed version of FFD (SIFFD) where deformations in certain regions of the moving image are constrained to be rigid [67]. The frameworks were the VoxelMorph (VXM) and segmentation-informed VoxelMorph (SIVXM) frameworks [37] and a joint registration and segmentation (JRS) framework [42]. Benchmarking was performed by comparing estimated displacement fields using the two metrics described in Section 3.2.

#### 3.4.1 Free-form deformation methods

Both FFD methods were implemented using NiftyReg version 1.5.39 [68]. The cost function consisted of three terms: a normalised mutual information term (NMI); a bending energy (BE) term and a term based on the symmetric and anti-symmetric parts of the Jacobian (LE) [68]. The full cost function was:

(2) $$C=\left(1-\lambda -\gamma \right)NMI-\lambda BE-\gamma LE$$

where λ and γ are cost weighting terms.

Three iteration levels were used in the optimisation of the cost function, with a maximum of 150 iterations in the final level. In SIFFD, deformations in the region of the image corresponding to the head segmentation estimated by the segmentation method were constrained to be rigid. While it may seem counterintuitive to use rigid constraints, the reason for using these was to prevent the pharyngeal wall (part of the head segmentation class) from being misregistered to the soft palate.

For both methods, several registrations were performed using different combinations of cost weighting term values and spline grid spacings (given in Supplementary Materials Table 1), and then evaluated using the metrics described in Section 3.2, enabling identification of the optimal values and spacings.

#### 3.4.2 VoxelMorph frameworks

The two VoxelMorph frameworks are almost identical; the only difference between them is the loss function used to train them. The SIVXM framework is trained using L (see Equation (1)), while the VXM framework is trained using a loss function consisting of two of the three terms in L:

(3) $$L_{\mathrm{VXM}}=MSE+\lambda {\left\|\nabla u\right\|}_{2}$$

The key difference between L and $L_{\mathrm{VXM}}$ is that the former contains a segmentation-dependent term (DSC). Use of L during training therefore results in a segmentation-informed registration framework, while use of $L_{\mathrm{VXM}}$ does not.

The frameworks were implemented in PyTorch 1.7.1 using the code publicly available at https://github.com/voxelmorph/voxelmorph. Framework training and evaluation was performed as described in Section 2.2.

The optimal learning rate and loss weighting term combination for each framework was identified via nested cross-validations as described in Section 3.3. Eight or more combinations (given in Supplementary Materials Table 1) were evaluated per framework.

#### 3.4.3 Joint image registration and segmentation framework

This framework was implemented in PyTorch 1.7.1 using the code publicly available at https://github.com/cq615/Joint-Motion-Estimation-and-Segmentation. The framework was trained in three stages using three different loss functions, as described in Section 2.2 of [42], and for 200 epochs in total. First, the registration CNN was trained for 67 epochs using $L_{\mathrm{VXM}}$ (see Equation (3)) as the loss function. Second, the segmentation CNN was trained for 67 epochs using cross-entropy (${\mathrm{CE}}_{pred\_seg}$) as the loss function. ${\mathrm{CE}}_{pred\_seg}$ was calculated by comparing the segmentations estimated by the segmentation CNN to the GT segmentations. Third, both CNNs were jointly trained for 66 epochs using a combination of $L_{\mathrm{VXM}}$, ${\mathrm{CE}}_{\mathrm{pre}d_{\mathrm{seg}}}$ and an additional cross-entropy term (${\mathrm{CE}}_{tra\_gt}$) as the loss function. ${\mathrm{CE}}_{tra\_gt}$ was calculated by comparing the fixed and transformed moving GT segmentations. The full loss function was:

(4) $$L_{\mathrm{JRS}}=MSE+\lambda {\left\|\nabla u\right\|}_{2}+\gamma_{1}{\mathrm{CE}}_{pred\_seg}+\gamma_{2}{\mathrm{CE}}_{tra\_gt}$$

where $\gamma_{1}$ and $\gamma_{2}$ are loss weighting terms. All other aspects of framework training and evaluation were performed as described in Section 2.2.

The optimal learning rate and loss weighting term combination was identified via a nested cross-validation as described in Section 3.3. Sixteen combinations (given in Supplementary Materials Table 1) were evaluated.

### 3.5 Ablation study

Although the segmentations consist of six classes, only the head, soft palate and vocal tract classes are required to determine if there is velopharyngeal closure. An ablation study was performed to investigate the effect of these three classes on the accuracy of the proposed framework. Three experiments were performed where different classes were used as inputs to the registration CNN during the training and evaluation of the framework. In the first, only the soft palate and vocal tract classes were used as inputs. In the second, the head, soft palate and vocal tract classes were used. In the third, all classes except the soft palate and vocal tract were used. In all other respects, the framework was trained and evaluated in the way described in Sections 2, 3.2 and 3.3.

### 3.6 Statistical tests

Groups of DSCs were compared using either a two-tailed Wilcoxon signed-rank test or a two-tailed sign test, depending on whether the distribution of differences between paired data points was symmetric. Groups of ASDs were compared in the same way as groups of DSCs. Numbers of true velopharyngeal closures were compared using McNemar’s test. A 5% significance level was used, corrected using the Holm-Bonferroni method to compensate for multiple comparisons.

## 4 Results

### 4.1 Optimal parameters and hyperparameters

Table 2 in the Supplementary Materials section lists the parameters identified as being optimal for the FFD methods and for training each framework.

### 4.2 Example images and segmentations

**Fig. 5:**
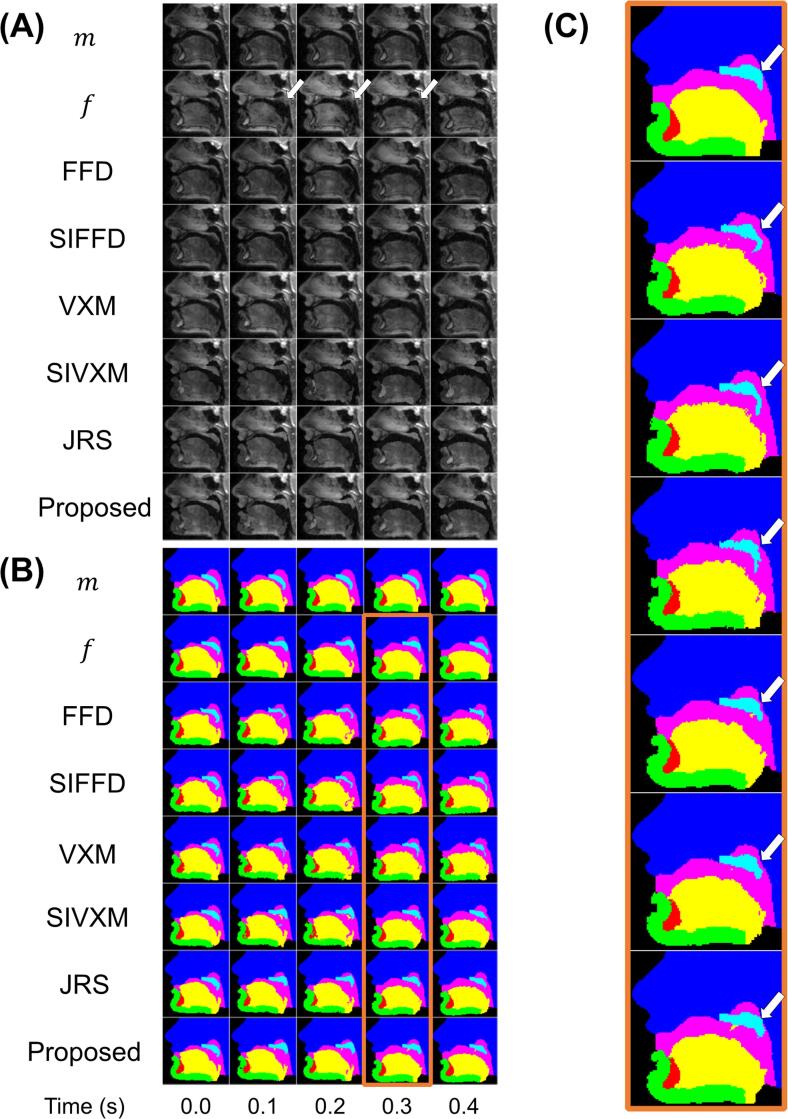
Transformed images and transformed ground-truth segmentations output by each method and framework, cropped to only show the vocal tract region. In (A), the first two rows show the moving image (m) and fixed image (f) pairs. The five fixed images are consecutive images from one of the image series and show a velopharyngeal closure. The white arrows show where the soft palate is in contact with the pharyngeal wall. The moving images are the reference image of the subject. The remaining rows in (A) show the transformed moving images output by the free-form deformations (FFD) and segmentation-informed FFD (SIFFD) methods and the VoxelMorph (VXM), segmentation-informed VXM (SIVXM), joint registration and segmentation (JRS) and proposed (Proposed) frameworks. In (B), the first two rows show the ground-truth segmentations of the moving image (M) and fixed images (F). The remaining rows in (B) show the transformed ground-truth segmentations output by each method or framework. (C) shows enlarged versions of the segmentations outlined in orange in (B). (For interpretation of the references to colour in this figure legend, the reader is referred to the web version of this article.)

Fig. 5 and videos included in the Supplementary Materials section show example transformed images and GT segmentations output by each of the methods and frameworks. In Fig. 5, the fixed images are consecutive images from one of the image series and show a velopharyngeal closure. This closure is captured by the proposed framework: contact between the soft palate and pharyngeal wall is shown in three of the transformed images and segmentations. However, the closure is not captured by the FFD methods or the VXM framework: none of the transformed images or segmentations show contact between the soft palate and the pharyngeal wall. The closure is partially captured by the SIVXM and JRS frameworks: two of the transformed images and segmentations output by the former framework show contact between the soft palate and the pharyngeal wall, while one of the transformed images and segmentations output by the latter framework shows such contact.

### 4.3 Displacement field accuracy evaluation

#### 4.3.1 Dice coefficients and average surface distances

**Fig. 6:**
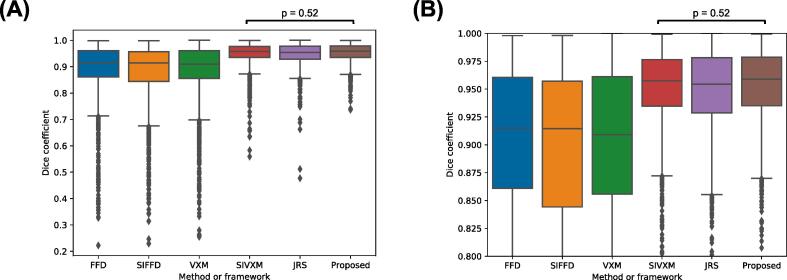
Dice coefficients (DSCs) of the transformed ground-truth segmentations output by the free-form deformations (FFD) and segmentation-informed FFD (SIFFD) methods and the VoxelMorph (VXM), segmentation-informed VXM (SIVXM), joint registration and segmentation (JRS) and proposed (Proposed) frameworks. The DSCs of all six classes are grouped according to framework only. (B) shows the section of (A) where the DSCs are between 0.8 and 1. There were statistically significant differences between all the DSC groups, except between the pairs of groups indicated with black bars in the boxplots.

**Fig. 7:**
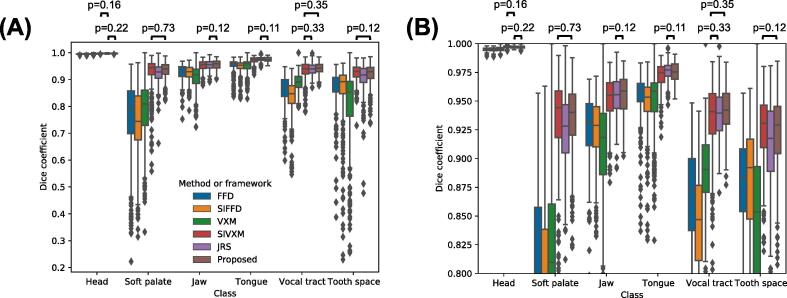
Dice coefficients (DSCs) of the transformed ground-truth segmentations output by the free-form deformations (FFD) and segmentation-informed FFD (SIFFD) methods and the VoxelMorph (VXM), segmentation-informed VXM (SIVXM), joint registration and segmentation (JRS) and proposed (Proposed) frameworks. The DSCs are grouped according to both framework and class. (B) shows the section of (A) where the DSCs are between 0.8 and 1. There were statistically significant differences between all the DSC groups, except between pairs of groups indicated with black bars above the boxplots.

**Fig. 8:**
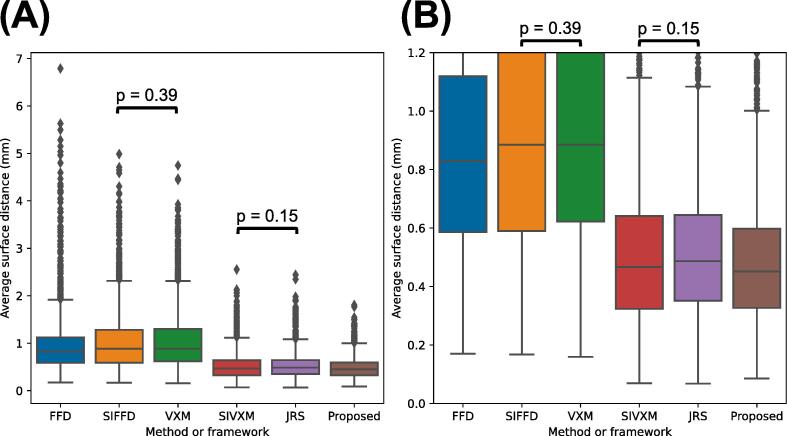
Average surface distances (ASDs) of the transformed ground-truth segmentations output by the free-form deformations (FFD) and segmentation-informed FFD (SIFFD) methods and the VoxelMorph (VXM), segmentation-informed VXM (SIVXM), joint registration and segmentation (JRS) and proposed (Proposed) frameworks. The ASDs of all six classes are grouped according to framework only. (B) shows the section of (A) where the ASDs are between 0.0 and 1.2. There were statistically significant differences between all the ASD groups, except between pairs of groups indicated with black bars above the boxplots.

**Fig. 9:**
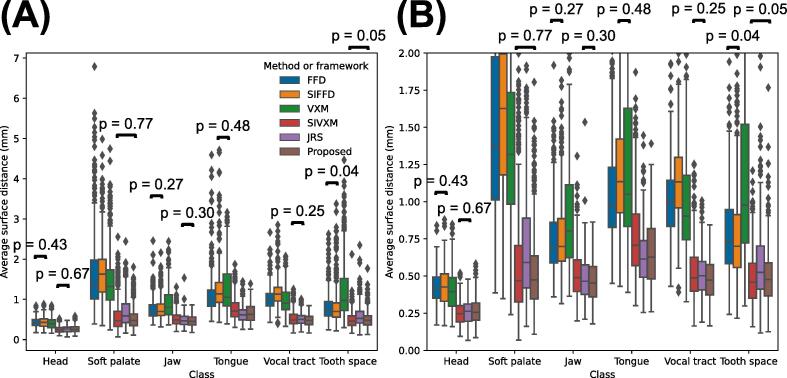
Average surface distances (ASDs) of the transformed ground-truth segmentations output by the free-form deformations (FFD) and segmentation-informed FFD (SIFFD) methods and the VoxelMorph (VXM), segmentation-informed VXM (SIVXM), joint registration and segmentation (JRS) and proposed (Proposed) frameworks. The ASDs are grouped according to both framework and class. (B) shows the section of (A) where the ASDs are between 0 and 2. There were statistically significant differences between all the ASD groups, except between pairs of groups indicated with black bars above the boxplots.

Fig. 6 and Fig. 7 show the DSCs of all classes in the transformed GT segmentations output by each of the methods and frameworks, while Fig. 8 and Fig. 9 show the ASDs of all classes.

As shown in Fig. 6 and Fig. 7, the median DSCs of the segmentation-informed frameworks were consistently higher than those of the FFD methods and VXM framework. There were statistically significant differences between the DSCs of these frameworks and those of the FFD methods and VXM framework.

No segmentation-informed framework consistently achieved statistically significantly higher DSCs than the others. Although the SIVXM framework achieved the highest median DSC in three classes (head, soft palate and tooth space), in two of these classes (soft palate and tooth space) there were no statistically significant differences between its DSCs and those of the proposed framework, and in the other class (head) there was no statistically significant difference between its DSCs and those of the JRS framework. Similarly, although the proposed framework achieved the highest median DSC in two classes (jaw and vocal tract), in one of these classes (jaw) there was no statistically significant difference between its DSCs and those of the JRS framework, and in the other class (vocal tract), there was no statistically significant difference between its DSCs and those of the SIVXM framework. However, the ranges of the DSCs of the proposed framework were consistently narrower than those of the other frameworks, suggesting improved robustness in registration performance.

As shown in Fig. 8 and Fig. 9, almost identical trends in framework performance were observed when the frameworks were evaluated using the ASD as when the frameworks were evaluated using the DSC.

#### 4.3.2 True velopharyngeal closures

**Fig. 10:**
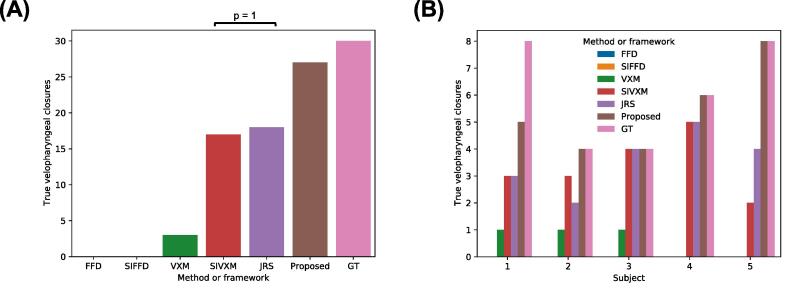
True velopharyngeal closures in the transformed ground-truth (GT) segmentations output by the free-form deformations (FFD) and segmentation-informed FFD (SIFFD) methods and the VoxelMorph (VXM), segmentation-informed VXM (SIVXM), joint registration and segmentation (JRS) and proposed (Proposed) frameworks. The bars labelled GT indicate the number of velopharyngeal closures in the GT segmentations of the fixed images. In (A) the true velopharyngeal closures are grouped according to framework only, while in (B) the true velopharyngeal closures are grouped according to both framework and subject. There were statistically significant differences between the true velopharyngeal closures captured by each framework, except between the frameworks indicated with the black bar in (A).

Fig. 10 shows the number of true velopharyngeal closures in the transformed GT segmentations output by each of the methods and frameworks.

The FFD methods failed to capture any velopharyngeal closures. Comparing the frameworks, the VXM framework captured the smallest number of velopharyngeal closures (3), while the proposed framework captured the largest (27). Furthermore, the proposed framework captured all the closures in four of the five image series, while the SIVXM and JRS frameworks only captured all the closures in one of the series and the VXM framework did not capture all the closures in any of the series. There were statistically significant differences between the true velopharyngeal closures captured by each framework, except between the SIVXM and JRS frameworks.

### 4.4 Ablation study

**Fig. 11:**
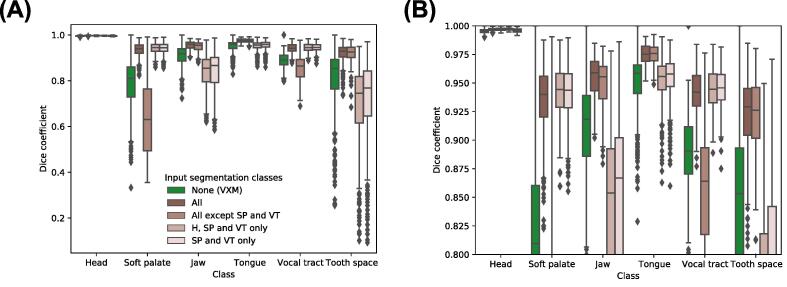
Dice coefficients (DSCs) of the transformed ground-truth segmentations output by the VoxelMorph (VXM) and proposed framework, grouped according to the segmentation classes of the transformed ground-truth segmentations (x-axis) and also the segmentation classes used as inputs to the registration convolutional neural network of the proposed framework during training and evaluation (colour code). In the Figure legend, ‘None (VXM)’ indicates the results of the VoxelMorph framework, ‘All’ indicates that all six segmentation classes described in Section 3.1.2 were used as inputs, while ‘H, SP and VT’ indicates the head (H), soft palate (SP) and vocal tract (VT) classes. (B) shows the section of (A) where the DSCs are between 0.8 and 1.

**Fig. 12:**
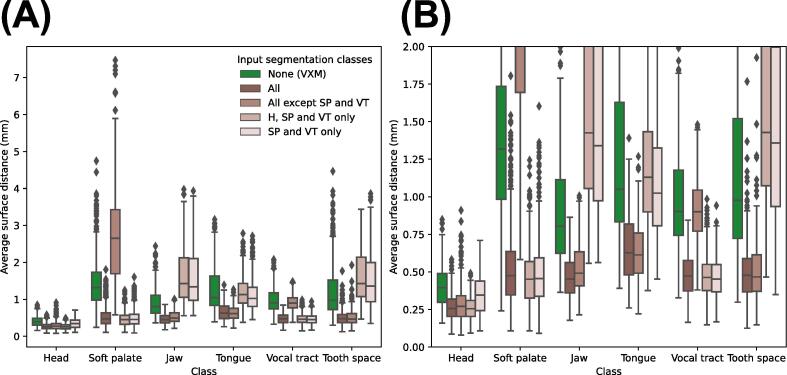
Average surface distances (ASDs) of the transformed ground-truth segmentations output by the VoxelMorph (VXM) and proposed framework, grouped according to the segmentation classes of the transformed ground-truth segmentations (x-axis) and also the segmentation classes used as inputs to the registration convolutional neural network of the proposed framework during training and evaluation (colour code). In the Figure legend, ‘None (VXM)’ indicates the results of the VoxelMorph framework, ‘All’ indicates that all six segmentation classes described in Section 3.1.2 were used as inputs, while ‘H, SP and VT’ indicates the head (H), soft palate (SP) and vocal tract (VT) classes. (B) shows the section of (A) where the ADSs are between 0 and 2.

Fig. 11 shows the DSCs of all classes in the transformed GT segmentations output by each version of the proposed framework, while Fig. 12 shows the ASDs of all classes. The median DSCs of the classes that were used as inputs to the registration CNN of the framework were consistently higher than those of the other classes, while the median ASDs of the classes were consistently lower.

**Fig. 13:**
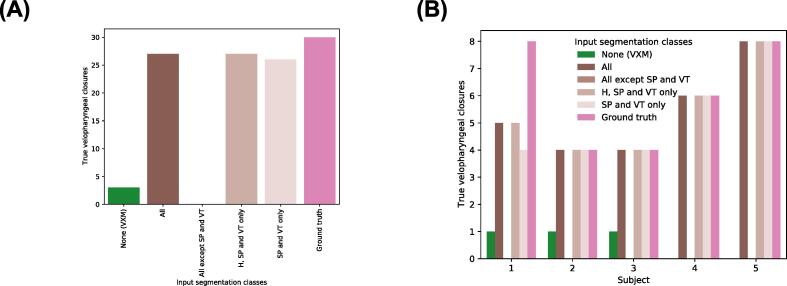
True velopharyngeal closures in the transformed ground-truth segmentations output by the VoxelMorph and proposed frameworks. The label ‘Ground truth’ indicates the number of velopharyngeal closures in the ground-truth segmentations of the fixed images. In (A) the closures are grouped according to the segmentation classes that were used as inputs to the registration convolutional neural network (CNN) of the proposed framework during training and evaluation. The label ‘All’ indicates that all six segmentation classes described in Section 3.1.2 were used as inputs, while ‘H, SP and VT’ indicates the head (H), soft palate (SP) and vocal tract (VT) classes. In (B) the true velopharyngeal closures are grouped according to subject (x-axis) and also the segmentation classes used as inputs to the registration CNN (colour code).

Fig. 13 shows the number of true velopharyngeal closures in the transformed GT segmentations output by each version of the proposed framework. The version where the head, soft palate and vocal tract classes were used as inputs to the registration CNN captured the same number of closures as the version where all classes were used as inputs, while the version where the soft palate and vocal tract classes were used as inputs captured one less closure. The version where the soft palate and vocal tract classes were not used as inputs failed to capture any closures.

## 5 Discussion

A framework for estimating displacement fields between dynamic 2D MR images of the vocal tract during speech was successfully developed. The framework is based upon the SIVXM framework [37] but features two adaptations. First, the framework includes a method to segment the images. Second, segmentations as well as images are used as inputs to the registration CNN, in the same manner as the framework of Chen et al. [43]. Incorporation of a segmentation method in the framework enables its use when segmentations of the images are not already available. This is the first time deep-learning-based deformable registration of MR images of speech has been investigated.

Evaluated using the DSC and ASD, the displacement field estimation accuracy of the proposed framework was superior to two FFD methods and a current state-of-the-art framework (the VXM framework), and very similar to two current state-of-the-art segmentation-informed frameworks (the SIVXM framework and a joint registration and segmentation framework). However, evaluated using a metric based on velopharyngeal closure, its performance was superior to all three state-of-the-art registration frameworks. In other words, the displacement fields estimated by the proposed framework captured more of the velopharyngeal closures in the image series, and therefore better captured this aspect of articulator motion than the other frameworks.

These results show that metrics based on clinically relevant and quantifiable aspects of organ motion can be used to evaluate the accuracy of registration frameworks and can be more sensitive to differences in accuracy than standard metrics such as the DSC and ASD.

In addition, these results show that registration CNNs input with segmentations as well as images can estimate displacement fields that better capture aspects of articulator motion than registration CNNs input with images only, even if the segmentations are estimates rather than ground truths.

The FFD methods failed to capture any velopharyngeal closures. This result is unsurprising as these methods are designed to estimate smooth and continuous displacement fields, while discontinuous displacement fields are required to capture the complex motion of the articulators. Removing the smooth and continuous displacement field constraints in the cost function did not improve the registration accuracy of the methods, showing that there are additional reasons why they are not appropriate for capturing articulator motion. When registering to fixed images showing velopharyngeal closure, the FFD method consistently misregistered the pharyngeal wall to the soft palate, instead of registering the soft palate to the soft palate. An example of this is shown in Fig. 5C. The SIFFD method, which ensured that the head (which includes the pharyngeal wall) deformed in a rigid manner, successfully prevented misregistration of the pharyngeal wall to the soft palate but did not improve the soft palate registration accuracy. Ideally, the proposed framework would have been compared with the FFD-based method developed by Hua et al. [33], as this method was designed to estimate displacement fields with discontinuities. However, unfortunately this was not possible as there is no publicly available implementation of the method.

The results of the ablation study show that unsurprisingly the head, soft palate and vocal tract segmentation classes are crucial for estimating displacement fields that accurately capture soft palate motion. This highlights the importance of using segmentations of the anatomical features whose motions are of interest but also segmentations of neighbouring features that provide information about the positions of the features of interest, for example whether the features of interest are in contact with other features. The results of the ablation study also show that using additional segmentation classes such as the jaw, tongue and tooth space did not affect the number of velopharyngeal closures captured by the framework. However, as shown in Fig. 11 and Fig. 12, using these additional classes was beneficial as it improved the accuracy with which they were registered by the framework.

To further encourage a CNN to estimate displacement fields that capture velopharyngeal closures, one approach for future investigation would be to use a loss function during CNN training that measures whether the starting points and durations of any velopharyngeal closures captured in a series of estimated displacement fields are correct. However, to be suitable for use in CNN training, this loss term would have to be differentiable. Developing a loss term that meets all these criteria would be challenging. A simpler approach would be to include a loss term based on whether individual transformed segmentations show contact between the soft palate and pharyngeal wall. This could be achieved using a topological loss term such as the one developed by [69] which can identify contact between different segmentation classes in a differentiable manner.

## 6 Conclusions

A framework for estimating displacement fields between dynamic 2D MR images of the vocal tract during speech was successfully developed and found to more accurately capture aspects of articulator motion than five current state-of-the-art deformable registration methods and frameworks. The framework is a step towards the ultimate goal of fully automatic quantification of articulator motion in such image series. In addition, a metric based on a clinically relevant and quantifiable aspect of articulator motion was proposed and shown to be useful for evaluating frameworks for registering dynamic MRI images of speech.

### CRediT authorship contribution statement

**Matthieu Ruthven:** Conceptualization, Methodology, Software, Validation, Formal analysis, Investigation, Resources, Data curation, Writing – original draft, Visualization, Project administration, Funding acquisition. **Marc E. Miquel:** Conceptualization, Methodology, Resources, Data curation, Writing – review & editing, Supervision. **Andrew P. King:** Conceptualization, Methodology, Writing – review & editing, Supervision.

## Declaration of Competing Interest

The authors declare that they have no known competing financial interests or personal relationships that could have appeared to influence the work reported in this paper.

## Data Availability

We intend to make the data used in this work publicly available.
